# Designing a Summer Transition Program for Incoming and Current College Students on the Autism Spectrum: A Participatory Approach

**DOI:** 10.3389/fpsyg.2018.00046

**Published:** 2018-02-13

**Authors:** Emily Hotez, Christina Shane-Simpson, Rita Obeid, Danielle DeNigris, Michael Siller, Corinna Costikas, Jonathan Pickens, Anthony Massa, Michael Giannola, Joanne D'Onofrio, Kristen Gillespie-Lynch

**Affiliations:** ^1^Department of Psychology, Hunter College, City University of New York, New York, NY, United States; ^2^College of Staten Island and the Graduate Center, City University of New York, New York, NY, United States; ^3^College of Staten Island, New York, NY, United States; ^4^University of Wisconsin–Stout, Menomonie, WI, United States; ^5^Lehman College, City University of New York, New York, NY, United States; ^6^Fairleigh Dickinson University, Madison, NJ, United States; ^7^Emory University School of Medicine, Atlanta, GA, United States

**Keywords:** autism spectrum disorder, disability, participatory research, self-advocacy, higher education

## Abstract

Students with Autism Spectrum Disorder (ASD) face unique challenges transitioning from high school to college and receive insufficient support to help them navigate this transition. Through a participatory collaboration with incoming and current autistic college students, we developed, implemented, and evaluated two intensive week-long summer programs to help autistic students transition into and succeed in college. This process included: (1) developing an initial summer transition program curriculum guided by recommendations from autistic college students in our ongoing mentorship program, (2) conducting an initial feasibility assessment of the curriculum [Summer Transition Program 1 (STP1)], (3) revising our initial curriculum, guided by feedback from autistic students, to develop a curriculum manual, and (4) pilot-testing the manualized curriculum through a quasi-experimental pre-test/post-test assessment of a second summer program [Summer Transition Program 2 (STP2)]. In STP2, two autistic college students assumed a leadership role and acted as “mentors” and ten incoming and current autistic college students participated in the program as “mentees.” Results from the STP2 pilot-test suggested benefits of participatory transition programming for fostering self-advocacy and social skills among mentees. Autistic and non-autistic mentors (but not mentees) described practicing advanced forms of self-advocacy, specifically leadership, through their mentorship roles. Autistic and non-autistic mentors also described shared (e.g., empathy) and unique (an intuitive understanding of autism vs. an intuitive understanding of social interaction) skills that they contributed to the program. This research provides preliminary support for the feasibility and utility of a participatory approach in which autistic college students are integral to the development and implementation of programming to help less experienced autistic students develop the self-advocacy skills they will need to succeed in college.

## Introduction

As increasing numbers of students with Autism Spectrum Disorder (ASD) enter college, they face unique challenges that impact their ability to succeed in college, including difficulties self-advocating, self-regulating, forging and maintaining social relationships, and taking care of daily needs amidst competing time demands (Vanbergeijk et al., 2008; Elias and White, 2017; White et al., 2017). The challenges autistic^1^ college students face are likely compounded by the fact that students with disabilities lose access to services that were previously available to them when they graduate from high school (IDEA, 2004^2^). Although college students are eligible to receive disability-related accommodations under the Americans with Disabilities Act (ADA, 2008^3^), they are confronted with the challenge of self-advocating to request needed accommodations, which they were not required to do in high school.

Education about self-advocacy is recommended by the National Technical Assistance Center on Transition (2017) as an essential aspect of supports to help students with disabilities more generally transition into adulthood. Nevertheless, high school curricula often focus on narrowly defined academic skills (e.g., grade point average) and fail to sufficiently address difficulties associated with autism, such as self-advocacy and social difficulties, that can impact academic functioning, and adaptation more generally, in college (Anderson and Butt, 2017). Previous research demonstrates that autistic high school students are less likely to be involved in their own transition planning than students with most other disabilities (Shogren and Plotner, 2012); only 2.6% of autistic students play a leadership role in their transition planning. As a result, college is often the first time that autistic individuals are accountable for knowing their rights, securing necessary accommodations, assuming a leadership role by educating others about their disability, and developing the self-knowledge and communication skills needed to accomplish these tasks (Test et al., 2005; Pillay and Bhat, 2012; Van Hees et al., 2015; White et al., 2017).

Although autistic self-advocates recommend that education to promote self-advocacy skills begin in childhood (Shore, 2004), self-advocacy interventions for autistic individuals remain scarce (Gillespie-Lynch et al., 2017a). Perhaps partially because they have not learned the link between self-advocacy and receiving needed accommodations, approximately one third of students who were identified as autistic in high school do not disclose this information in college (Newman et al., 2011). Moreover, college instructors may also lack knowledge about autism (Pillay and Bhat, 2012). Consequently, autistic students who do not know why or how to self-advocate may not receive needed accommodations in college.

Even though prior research demonstrates a clear need for programming to help autistic college students transition into and succeed in college (Glennon, 2001; Adreon and Durocher, 2007; Hendricks and Wehman, 2009; Kapp et al., 2011; Pillay and Bhat, 2012; Gobbo and Shmulsky, 2014) and the Interagency Autism Coordinating Committee (2013) identified transition programming for autistic adults as a priority, only a very small body of research has provided preliminary evidence that supports for incoming and current autistic college students are attractive to and/or beneficial for them (Pugliese and White, 2014; Schindler et al., 2015; Ames et al., 2016; Barnhill, 2016; White et al., 2016a, 2017; Gillespie-Lynch et al., 2017a,b; Roberts and Birmingham, 2017; Hillier et al., 2018). As an early example of this type of work, Pugliese and White (2014) demonstrated the feasibility and acceptability of a cognitive-behavioral group-based problem solving skills intervention for five autistic college students. Eleven autistic college students who participated in a mentorship program delivered by occupational therapy students reported improvements in occupational performance, e.g., time management, organization of assignments, and socialization with peers (Schindler et al., 2015). Similarly, twelve autistic college students who completed evaluations of a structured mentorship program reported satisfaction with programming and success in obtaining goals (Ames et al., 2016). Qualitative coding of interviews conducted with nine autistic college student mentees and nine mentors revealed that mentees more successfully self-advocated during mentorship as they gained experience with their mentor; that they enjoyed having a familiar, supportive connection in their mentor; and that mentors and mentees learned together through the mentorship process (Roberts and Birmingham, 2017). Twenty-six autistic college students who completed evaluations after participating in one of nine support groups with a structured curriculum over the course of 6 years of programming reported reduced anxiety and loneliness and increased self-esteem (Hillier et al., 2018).

Although the growing emergence of programs to support autistic college students is promising, existing supports are often not well informed by the skills and perspectives of the students they are designed to serve (Barnhill, 2016). Therefore, we used a *participatory approach*, wherein autistic college students played a leading role in developing and administering supports, to develop programming to help autistic students transition into and succeed in college. This approach to program development is grounded in *positive psychology*, which emphasizes building from existing strengths rather than focusing on deficits. Central to this perspective is the concept of *self-determination*, i.e., “people with and without disabilities can become causal agents in their own lives…as long as they are provided the opportunities and supports necessary to develop and express these skills and attitudes” (Shogren et al., 2006, pp. 338–339). Self-determination, comprised of several components, including independence, self-advocacy, self-efficacy, and self-management, is a key predictor of a “successful transition to adult life” (Malian and Nevin, 2002, p. 73). We hoped that a participatory approach to developing supports for transitioning and current autistic college students would increase self-determination by providing opportunities for current autistic college students to demonstrate agency and serve as role models for less experienced autistic students while enriching program design through insights derived from the lived experience of being an autistic college student.

Although sophisticated participatory approaches to developing supports for autistic individuals have been developed (e.g., Nicolaidis et al., 2011), participatory designs are not widely used in autism research. A recent review identified only seven studies that described participatory research partnerships between academic researchers and individuals with ASD or other neurodevelopmental disorders (Jivraj et al., 2014). Indeed, participatory research with autistic individuals lags behind research with individuals with intellectual disabilities (Shogren et al., 2006). This gap in the literature is surprising given that autism is associated with a number of strengths that are beneficial for research including honesty, detail orientation and a systematic approach to knowledge production (e.g., Baron-Cohen et al., 2009).

In order to foster self-determination, we offer an ongoing participatory mentorship program (Project REACH), wherein autistic college students and students with other disabilities help develop programming and become mentors in the program. The development of the transition program reported in the current paper was intended to advance participatory supports for this population beyond our existing program. As context, our mentorship program was developed by the last author of this report and is reported on elsewhere (Gillespie-Lynch et al., 2017a). Participation in initial iterations of this program was associated with reduced self-reported anxiety and autism symptoms among 28 students with disabilities (12 of whom were autistic) and increased academic self-efficacy, self-advocacy knowledge, and perceived social support among 30 students (17 of whom were autistic; Gillespie-Lynch et al., 2017a).

As a rare exception to the lack of available participatory research for autistic college students, White and colleagues have also been developing a participatory line of research to develop services for transitioning and current autistic college students. In a recent paper, White et al. (2017) described an iterative process for developing transition supports for autistic high school and college students that is somewhat similar to the process we will describe in this report. Guided by a needs assessment focus group with autistic college students, White and colleagues developed and piloted two potential interventions, a virtual reality intervention (*n* = 4) and an in-person cognitive behavioral therapy (CBT) intervention (*n* = 4). Participants indicated that the in-person CBT training (average rating of 6.5 out of 10) and the virtual reality intervention (average rating of 4.75 out of 10) were moderately helpful with respect to a range of outcomes, including monitoring progress, increasing awareness of communication skills, and addressing participants' self-identified goals (White et al., 2016a). White and colleagues then conducted an online survey and focus groups with autistic adolescents and young adults (focus group: *n* = 5; survey: *n* = 5), parents (survey: *n* = 32), and professionals who worked with autistic people in high school or college (focus groups: *n* = 10; survey: *n* = 20) to gather diverse stakeholders' insights about challenges associated with ASD and existing supports (White et al., 2016b).

Utilizing insights derived from this research and a subsequent set of focus groups with secondary and postsecondary educators (Elias et al., submitted), White and colleagues developed a transition program, the *Stepped Transition in Education Programming for Students with ASD* to support autistic high school students (STEPS 1) and college students (STEPS 2) and adapted it utilizing feedback from clinicians, educators, parents, and autistic students. Although the use of multiple stakeholders to inform intervention design is a significant strength of their approach, the report describing this participatory process does not contain information about the number of stakeholders who provided feedback on curriculum design or the nature of their feedback (White et al., 2017). Correspondence with the authors revealed that two autistic college students provided feedback on the program via email and telephone.

Students participating in the STEPS curriculum receive *individualized* online and in-person supports through bi-weekly counseling sessions with a counselor, parent and/or trusted school personnel. The STEPS program is currently being evaluated; initial feasibility data reveals substantially higher satisfaction ratings among 26 autistic students (*M* = 4.31 out of 5) than were obtained for White and colleagues' initial CBT and virtual reality interventions prior to adaptation. Although individualized supports, such as the STEPS program, are a necessary aspect of treatment for autistic individuals given the diversity of the spectrum, activities with peers may be particularly beneficial for youth with disabilities. For example, participation in a group afterschool program for adolescents with ADHD was associated with sustained improvements in organizational skills, academics and inattention relative to participation in a similar in-school program comprised of one-on-one meetings with an adult mentor (Evans et al., 2016).

In response to the need for participatory transition programming that directly aligns with the needs and interests of autistic college students, the current study utilized a participatory approach to develop a manualized self-advocacy-focused summer transition program, wherein students on the spectrum were integral to the development and modification of the program and corresponding manual and played a guiding role in helping incoming autistic students learn the self-advocacy skills needed to transition into and succeed in college. This program was primarily focused on self-advocacy and related constructs (e.g., self-efficacy, autism knowledge, and disability identity), but also included support for fostering interrelated skills and knowledge, including social skills and classroom readiness. Consistent with recommendations from experts in participatory research about autism (Nicolaidis et al., 2011), we provide detailed information about the process of involving autistic students in program design and key insights they generated.

The following research questions were addressed:

1. What is the feasibility of a participatory approach wherein autistic college students play a leadership role in program design and implementation of a summer transition program?
2. Is participation in a transition program associated with enhanced self-advocacy skills (including improved knowledge about ASD, disability identity, and disclosure), enhanced academic self-efficacy and/or reduced self-reported ASD symptoms?
3. What recommendations for future programming can be derived from this participatory approach?

## Methods

### Overview

**Figure 1:**
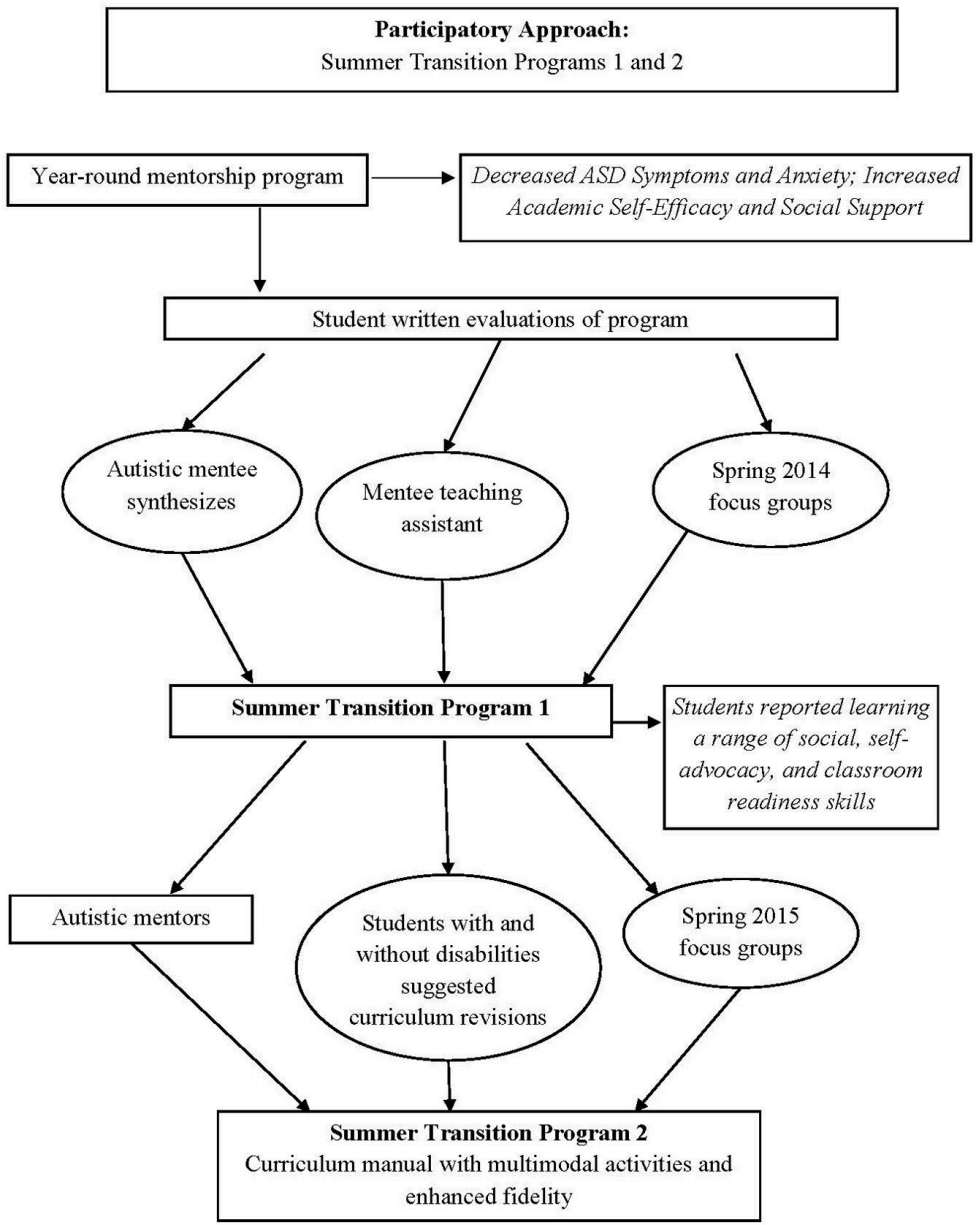
Participatory approach for summer transition programs 1 and 2.

We utilized an iterative participatory approach to develop a no-cost summer transition program for incoming and current autistic college students (illustrated in Figure 1). The iterative process of the current research occurred as follows. First, we adapted the curriculum of our semester-based participatory mentorship program, wherein autistic college students and students with other disabilities help develop programming and become mentors in the program, developed by the last author of this report. The process of adapting the mentorship program was guided by recommendations from an autistic student who synthesized his peers' evaluations of the mentorship program, in addition to recommendations from prior literature. Following the adaptation of the mentorship program curriculum, we conducted an initial feasibility assessment of the summer transition program in 2014 [Summer Transition Program 1 (STP1)]. We then developed a curriculum manual by revising our initial curriculum guided by data and extensive feedback from two independent study students (another autistic student and her mentor with a different disability). Finally, we pilot-tested the second summer transition program in 2015 wherein we utilized the manualized version of the curriculum [Summer Transition Program 2 (STP2)]. STP1 was primarily conducted as an initial feasibility assessment to inform STP2, which pilot-tested the manualized version of the curriculum. As such, results are reported in greater detail for STP2. This research was carried out in accordance with the recommendations of our college Human Research Protections Programs with written informed consent from all subjects.

### Iterative process of developing a summer transition program curriculum

The summer transition program overarching model and curriculum were developed for an initial feasibility assessment of the program (STP1), and subsequently adapted for the pilot-test (STP2) based on lessons learned and feedback from program participants (described in detail in subsequent sections). The program model was guided by available interventions to support social and self-advocacy skills (e.g., Paradiz, 2009; Laugeson et al., 2012) and recommendations from mentors and mentees.

The program was structured as a weeklong summer program comprised of 5 h of instruction and recreation across 5 days. On the fifth day, students participated in a pizza party and completed post-tests. Many aspects of the program were modeled after a typical college classroom environment, including 45-minute lectures with small breaks between each lecture. Classroom-based trainings were facilitated by doctoral-student researchers and supported by a team of undergraduate and graduate-student volunteers. Workshop facilitators led each training module by discussing a skill, demonstrating the appropriate use of the skill, and then asking each student to use the skill (i.e., role-play) with a peer. As a core component of the mentorship model, students were encouraged to practice their skills with their peers, mentors, volunteers, and workshop facilitators. This allowed students to practice their skills in a supportive environment, with constructive feedback from their peers and facilitators. Students also engaged in games to help them interact with one another and feel more engaged, such as a scavenger hunt to learn about on-campus resources. We relied on a scaffolding approach to learning (Wood and Middleton, 1975) in which the first few days of the program presented basic skills; later in the program, students were asked to build upon these skills. Scaffolding was provided via a team of undergraduate mentors and doctoral student facilitators who provided individualized support to participants throughout the program. The last author supervised the summer programs.

The program curriculum for the first summer transition program was adapted from our ongoing, peer-mentorship program for autistic college students and students with other disabilities (Gillespie-Lynch et al., 2017a,b). Students in our mentorship program are invited to attend weekly individualized one-on-one and/or group mentorship sessions with a structured curriculum throughout the school year. A central focus of our mentorship program is on helping students develop self-advocacy skills through a structured curriculum with opportunities to practice skills through games and role-plays and by encouraging them to take on leadership roles in the program as mentors and/or researchers. Mentees who are transitioning into becoming mentors are paired with a more experienced mentor who attends their one-on-one meetings with their mentee and provides constructive feedback after each meeting. We adapt the mentorship curriculum each term after providing students with an overview of prior curriculum and asking for their ideas about what they would like to learn about. Over the past 5 years, the curriculum has addressed social skills, self-advocacy skills, executive function and self-regulation skills, interview and employment readiness skills, and writing skills.

In order to guide us in preparing curriculum for the first summer transition program, an autistic mentee conducted independent study research synthesizing end-of-the-term program evaluations provided by 20 mentees in our year round program, prior literature, and his own experiences in a final paper wherein he articulated several recommendations to better support transitioning autistic students. His recommendations included:

“*Teaching about self-advocacy… [including] taking responsibility, making decisions that will affect your life, and improving your life,”* emphasizing “*the importance of choosing your classes carefully… [so they match] your interests.”* He also discussed “*the importance of ensuring your professor is aware of your disability as quickly as possible,”* to provide opportunities for “*new students to be able to interact with others who are similar to them [during]… group activities,”* and to “*find ways to keep students invested in each lesson.”* He recommended a range of engaging activities, as it is “*easier to learn by doing than it is to do so by merely listening.”*

This student later went on to participate in our first summer transition program. Although he was academically gifted, he had behavioral issues which almost caused him to be asked to leave college when he first began. Observing the challenges he faced adapting to college, we decided that more intensive programming than the 2 h per week available through our mentorship program was needed for students facing heightened difficulties adapting to college and that such programming would be most beneficial *before* students started college. Therefore, this student was both an impetus for and a co-designer of programming he then participated in.

We also convened a focus group led by five facilitators that involved approximately ten mentors and mentees from the year-round mentorship program to identify curriculum topics for the transition program. Focus group participants were informed of the upcoming summer program and asked to use their experiences in the mentorship program to generate recommendations about the types of programming incoming students on the spectrum would benefit from. The focus group resulted in a detailed list of potential curriculum topics, activities, assessments, and recommendations for how to make programming engaging for students. A student volunteer, who had previously been a mentee in the mentorship program, played a significant role in assisting with the development of the curriculum materials for the first summer program. He also served as a teaching assistant and peer mentor during the program, provided a synthesis of student reflections on the program, and is one of the authors of this report.

### Summer transition program assessment approach

The same assessment approach was applied to both STP1 and STP2, with additional assessments conducted during STP2 to gain a greater level of insight into the program (described in detail in the STP2 section of this manuscript). Online pre/post-surveys administered via SurveyMonkey included the following measures: (1) the Social Responsiveness Scale-A (Constantino and Gruber, 2012); (2) the Disability Identity Scale (Darling and Heckert, 2010); (3) an Academic Self-Efficacy Scale (Hoover-Dempsey and Sandler, 2005); and an adapted version of the Autism Awareness Survey (Stone, 1987; Gillespie-Lynch et al., 2015). Anxiety was also assessed but is not reported here due to an error in the instructions for the survey. The Test of Nonverbal Intelligence (Brown et al., 1997) and structured interviews were administered in-person.

#### Academic self-efficacy scale

The *Academic Self-Efficacy Survey* (Hoover-Dempsey and Sandler, 2005) measured students' beliefs in their academic abilities. They were asked to rate their perceived ability to learn the things taught in school, do even the hardest homework if they try, and figure out difficult homework on a 4-point scale from 1 (not true) to 4 (very true).

#### Autism awareness scale

An adapted version of the *Autism Awareness Survey* (Stone, 1987; Gillespie-Lynch et al., 2015) was used to assess knowledge about autism. Item responses were given on a 5-point Likert scale and then summed to provide a total autism knowledge score.

#### Disability identity and opportunities scale

The *Disability Identity and Opportunities Scale* (Darling and Heckert, 2010) was used to explore students' disability pride, feelings of exclusion, orientations toward the medical model, or the view of disabilities as abnormalities within individuals that are in need of correction (Humphrey, 2000), and orientations toward the social model, or recognition of society's role in constructing disability (Oliver, 1990). Sample items include, *I wish someone would find a cure for my disability* (medical model subscale) and *I am familiar with the Disability Rights Movement and support its goals* (social model subscale). Higher scores indicate heightened alignment with each construct. Moderate to excellent internal consistency was demonstrated for the total measure (*alpha* = 0.81), as well as for the subscales (pride: *alpha* = 0.91; exclusion: *alpha* = 0.50; medical: alpha = 0.52; social: *alpha* = 0.62).

#### Social responsiveness scale-A

The *SRS-A* (Constantino and Gruber, 2012) is an adult self-report measure of autism symptoms comprised of five subscales that include Social Awareness, Social Cognition, Social Communication, Social Motivation, and Restricted Interests and Repetitive Behaviors. Prior research has shown that the social responsiveness scale is internally consistent with strong construct validity (Constantino and Gruber, 2012). Higher scores indicate heightened autistic traits.

#### Test of nonverbal intelligence–third edition

Nonverbal intelligence was measured with the *Test of Nonverbal Intelligence*, wherein participants are asked to complete increasingly complex visual puzzles (TONI-3; Brown et al., 1997). The TONI-3 was administered once during post-testing. The TONI-3 is often favored over other intelligence tests because cultural and educational backgrounds have not been found to affect test results (Brown et al., 1997).

#### Semi-structured interview

A researcher-developed interview was conducted at pre- and post-test to assess participants' needs and potential gains pertaining to self-advocacy-related knowledge and skills. Interview scripts are available upon request from the last author. Data were analyzed using a grounded theory approach to develop themes for coding (Draucker et al., 2007). A coding dyad reviewed all of the responses to a given question, developed themes that reflected the majority of responses, and then collaboratively coded a subsection of responses (approximately 2 responses). Each member of the dyad then independently coded all of the remaining responses from a given prompt. Coding from each member was compared to evaluate reliability. This form of consensus coding was used throughout the open-ended responses found in the survey and interview data. Codes developed were not mutually exclusive and reliability for each code was 80% agreement or higher.

#### Analytic approach

We utilized a mixed methods approach whereby qualitative and quantitative assessments mutually informed iterative curriculum development (Yoshikawa et al., 2008). Primary quantitative analyses included descriptive statistics and nonparametric tests to identify pre/post-testing trends. Primary qualitative analyses were utilized to address the primary research questions with a greater level of depth and nuance not available through the quantitative measures.

### Summer transition program 1 (STP1)

The first summer transition program (i.e., Summer Transition Program 1; STP1) was conducted as an initial feasibility assessment of the program. The program took place the week of 8/18/14 for 5 h every weekday (see Appendix A for the program syllabus).

#### STP1 participants

Participants were recruited via the Center for Student Accessibility at a non-selective urban college in an outer borough of New York City and at open houses held by the college for incoming freshmen. Researchers also provided flyers to counselors at local high schools. Participation was free of charge and students received $50 in Amazon gift cards for completing pre- and post-testing. Recruitment efforts resulted in a sample of 14 students (eleven transitioning and three returning students) who took part in the pre-testing phase of the study. Thirteen students decided to participate in the transition program; pre-test data includes one student who decided not to participate in the summer program due to scheduling issues and instead joined the mentorship program in the fall. One student did not complete a pre-test interview due to scheduling issues. Three students did not complete post-test interviews, two due to scheduling issues and one (described below) had pronounced co-occurring mental health issues and preferred to talk about personal concerns rather than answering structured interview questions.

Students ranged in age from 17 to 28 years old (*M* = 19.07; *SD* = 2.76; see Table 1 for participant characteristics). The majority of the students were male (*n* = 12; 87.5%) and White (*n* = 10; 71.4%). Twelve students provided official documentation of their ASD diagnosis. Of the two students who did not provide written documentation of having ASD, one was a sophomore whose IEP indicated that he had attended an autism specific high school; both he and his mother reported that he had ASD. The other student who did not provide documentation completed half of the pre-test interview and took the post-test survey, but did not complete the pre-test survey and post-test interview due to difficulties with emotion regulation. Although she did not self-identify as autistic during the program, both she and her mother later reported that she was autistic when she participated in our year-round mentorship program. On average, scores on the TONI-3 indicated that students had nonverbal intelligence levels in the typical range (i.e., index scores > 90), with pronounced variability (*M* = 99.82, *SD* = 15.61). Additionally, participants self-reported moderate symptoms associated with ASD on the SRS-A at pre-testing, also with pronounced variability (*M* raw score = 69, *SD* = 20.27). All participants were able to communicate through spoken language fluently.

**Table 1:** Participant characteristics: summer transition program 1.

**Variable**	**% or *M* (*SD*)**
Gender	
% Male	85.7%
% Female	14.3%
Race	
% White	71.4%
% Black/African American	14.3%
% Asian	7.1%
% Did not identify	7.1%
Ethnicity	
% Hispanic	14.3%
% Non-Hispanic	85.7%
Provided documentation of ASD diagnosis	
% Yes	85.7%
% No	14.3%
Mean Age	19.07 (2.76)
Mean TONI Score	99.82 (15.61)
Mean Pre-Test SRS	68.67 (20.27)

*Documentation of ASD diagnosis included written documentation to the Center for Student Accessibility indicating that the student had received an educational determination of autism and/or a clinical diagnosis of ASD*.

### STP1 results

#### Preliminary results

When students were asked during pre-test interviews whether high school had generally prepared them for college, nine indicated that it had and three reported that it had not (one student was not asked this question and one student did not complete a pre-test interview as described above).

#### Is participation in a transition program associated with enhanced self-advocacy skills (including improved knowledge about ASD and disability identity), enhanced academic self-efficacy and/or reduced self-reported ASD symptoms?

No changes in standardized measures from pre-test to post-test were observed (*p*s > 0.09; the only statistical trend was toward *increased* feelings of exclusion following participation). However, when asked during post-test interviews if they had learned anything from the program, students reported learning a range of social (*n* = 9), self-advocacy (*n* = 7), and classroom readiness skills (*n* = 4). Two students stated that they learned “*Self-advocacy mostly; stress skills and all; how to handle disagreements.”* and “*I have learned how to note take. I have learned how to self-advocate for myself. And I've learned a great deal about socialization too.”* An autistic student with a co-occurring intellectual disability said “*I learned about my rights as a person with disabilities and…that there's nothing wrong with having a disability and that there's others out there with a disability and there's always people willing to help with whatever you need. And I learned that, I learned how to enter a conversation and hold a conversation.”* When asked at pre-test to define self-advocacy, eight students said that they *didn't know* and four said standing up for yourself, one also said standing up for others (one student was not asked this question). When asked to define self-advocacy at post-test, all nine of the students who were asked this question said standing up for yourself (one also said standing up for others).

### Key recommendations from summer transition program 1

Findings from this initial feasibility assessment provided several recommendations for future programming that we utilized when developing the curriculum manual evaluated during the second summer transition program:

1. Future programming should continue to offer autistic students the opportunity to serve in leadership and development roles. The impetus for the mentor/mentee setup in the second summer transition program was spurred by the integral role of the teaching assistant who had been a mentee. The teaching assistant was uniquely positioned to liaise between students and program leaders and in effect served as a valuable resource for providing a consistent feedback loop between participants and program leaders.
2. Qualitative data provided invaluable insights about the experiences and perspectives of program participants. Interviews should be adapted to more fully allow students to describe their experiences in the program.
3. In order to continue to build on the recommendations of the autistic independent study student who recommended that we “*find ways to keep students invested in each lesson,”* programming should utilize more multi-modal forms of instruction to better engage students with diverse learning styles and preferences. Lectures, media clips, role-plays, hands-on activities, and small- and large-group activities should be used to encourage participation from students who may not learn best from more typical classroom lectures. As an example of diverse preferences, many students reported that they specifically enjoyed the media clips that were embedded in many of the lectures whereas others expressed a preference for the outdoor activities. In addition, the teaching assistant highlighted that program participants particularly enjoyed the games embedded within the program, but struggled to engage in more group-oriented activities where students were asked to self-reflect. These small group environments may have helped students to build their social skills, but may have also increased students' anxiety as a result of forced social interaction and need for self-reflection. To address this limitation, we invited a combined theater and psychology honors student (the eighth author) to be the “games master” for the second summer transition program to help us develop theater-based games to more effectively engage shyer students.

### Revising and adapting the summer transition program

In order to begin the process of developing and pilot-testing STP2, two independent study students with disabilities (an autistic student who would become a mentor in the second summer transition program and her mentor who had a different disability) provided feedback on the curriculum developed for the first summer transition program as well as suggestions for improvement. The autistic independent study student wrote an independent study paper entitled “*Steps toward Improving a Summer Transition Program for College Students on the Autism Spectrum: A Focus on Self-Advocacy.”* In her paper she stated:

*While the Summer Transition Program is a great program, it still needs room for improvement. In some of the modules it would be beneficial to introduce the incoming students on the importance of self-advocacy…We can also teach them more about exercising self-awareness with themselves and what is around them (mindfulness training) in addition to working on strategies on how to develop effective studying skills and ways to lessen anxieties for exams or schoolwork….Another recommendation that I would suggest for the summer transition program is to have it become more engaging and reduce the amount of text used in the modules or power points and make them more self-explanatory such as using images or doing an activity. Instead of having the meetings become a lecture or just presenting slides we can make it more interactive and have the students provide feedback if needed*.

We invited an additional autism specialist with extensive experience in intervention design (the fifth author) and additional doctoral students with prior experience with autism research and/or service (the third, fourth, and seventh authors) to join our research team and help us develop a curriculum manual. We utilized findings from the first summer transition program and feedback from the aforementioned independent study students and from a second focus group, comprised of 7 autistic mentees, 2 students with other disabilities, 3 mentors without disabilities, 4 doctoral students and the last author, to develop the curriculum manual for the second summer transition program.

Our curriculum manual is available open access: (https://www.researchgate.net/publication/294261324_Building_Bridges_for_Autistic_College_Students_Project_REACH_Summer_Transition_Program_Manual).

### Summer transition program 2 (STP2)

In accordance with our program orientation that emphasizes the leadership of autistic students, the pilot-test of the program was led by a collaboration of two psychology professors and autism specialists, four psychology doctoral students, as well as a team of five undergraduate *mentors* (two transitioned autistic mentees from the mentorship program and three undergraduate students without ASD). The two autistic mentors (a male and a female, both Caucasian) provided documentation of their autism diagnoses.

During the second summer transition program, the last author utilized the fidelity of delivery ratings outlined in our manual to score the consistency with which instructors administered the planned curriculum during each session along with a shifting array of other in-person fidelity raters. Given that the other in-person raters shifted throughout each day, the first and last author coded videotapes of modules delivered by each of the instructors to verify that fidelity measures could be attained reliably and obtained reliability of 100%.

#### STP2 participants

Recruitment efforts were diverse, and included outreach to a range of high schools and colleges throughout the country. Students planning to attend or attending any college were eligible to participate. We conducted the second summer transition program the week of 08/03/15 at a fairly selective college in Manhattan (to be accessible to students throughout the NYC area; see Appendix A for the program syllabus). Ten students participated in the second transition program: two were in their junior year of high school, six were transitioning into college and two were continuing their college careers (one of these students was transitioning from a 2 to 4-year college while another student had been facing pronounced challenges in his college). Surprisingly, none of the students were planning to attend the college where the program was located.

Mentees ranged in age from 17 to 22 years old (*M* = 18.8, *SD* = 1.58; See Table 2 for participant characteristics). The majority of the mentees were male (*n* = 8; 80%). With respect to race and ethnicity, the mentees identified as Caucasian (*n* = 5), Mixed (*n* = 3), Black (*n* = 1), and Chinese (*n* = 1). All participating mentees provided official IEP documentation of a disability and all of their parents identified them as having ASD. Eight out of ten IEPs specified ASD or PDD-NOS, while two IEPs did not identify a specific disability. However, one student with an IEP that did not mention any disability classification had traveled from Ohio to NYC specifically for our program. The other student brought her IEP from the residential facility where she was living and the page specifying diagnosis was missing. Numerous attempts to obtain verification from these students and their parents were unsuccessful. However, both of these students self-identified as autistic.

**Table 2:** Mentee characteristics: summer transition program 2.

**Variable**	**% or *M* (*SD*)**
Gender	
% Male	8 (80%)
% Female	2 (20%)
Race	
% White	6 (60%)
% Black/African American	2 (20%)
% Asian	2 (20%)
Ethnicity	
% Hispanic	2 (20%)
% Non-Hispanic	8 (80%)
Provided documentation of ASD diagnosis	
% Yes	8 (80%)
% No	2 (20%)
Education Status	
% Transitioning	6 (60%)
% Current college student	2 (20%)
% High school students	2 (20%)
Mean Age	18.50 (1.58)
Mean TONI Score	97.30 (7.44)
Mean Pre-Test SRS	67.00 (28.09)

On average, TONI-3 scores indicated that students had nonverbal intelligence levels consistent with their age (index scores > 90), with some variability (*M* = 97.30, *SD* = 7.44). Additionally, participants reported moderate symptoms of ASD on the SRS-A at pre-tests, with substantial variability (*M* raw score = 67, *SD* = 28.09). All participants were able to communicate through spoken language fluently. No differences in nonverbal IQ or autism symptoms were observed between participants in the first and second summer transition program (*p*s > 0.25).

#### STP2 measures

The measures described above for the evaluation of STP1 were utilized again for the current study. The semi-structured interview was adapted for STP2. We also added follow-up interviews to gain additional insight into program impacts.

##### Semi-structured interviews

The semi-structured interview used during the first summer transition program was adapted to elicit more elaboration; it included questions that invited the participant to discuss their general expectations (pre-test) and reactions (post-test) pertaining to the program, as well as to (1) define self-advocacy, (2) discuss strengths their disability affords them, (3) demonstrate how they would secure accommodations in college, and (4) describe the process of evaluating if and when to disclose. One student was not asked questions about self-advocacy at pre-test due to interviewer error. His responses to these questions are also not reported at post-test to keep pre-test and post-test data comparable.

Semi-structured interviews were conducted with mentors from the program 1 week after the summer transition program. Two mentors (both without ASD) declined participation in these interviews. Interviews included questions assessing: (1) perspectives and strategies that mentors shared with mentees; (2) what they learned from being mentors, (3) how mentors utilized self-awareness skills and whether they experienced any challenges relating to self-awareness; (4) mentors' understanding of mentees, including any challenges they encountered related to understanding, connecting with, and setting boundaries with mentees; (5) mentors' understanding of autism, (6) mentors' views on self-advocacy; and (7) mentors' perspectives on their roles as mentors and the summer transition program more generally.

##### Follow-up interviews

As an addition program evaluation strategy, we reached out to all mentees 6 months after the program ended to see if they would like to participate in follow-up interviews. Interviews were adapted from the pre/post interview. Four mentees chose to participate in follow-up interviews. They were asked to reflect on their perspectives and experiences as well as skills fostered through their participation in the program.

### STP2 results

#### Preliminary results

When asked to describe expectations for the program, nine out of ten participants referenced learning general skills and knowledge (e.g., “*what to do, what not to do”*), with half of the participants mentioning specific college-related skills (e.g., “*skills for college to help me succeed”*). No participants specifically mentioned learning about self-advocacy.

#### Is participation in a transition program associated with enhanced self-advocacy skills (including improved knowledge about ASD and disability identity), enhanced academic self-efficacy and/or reduced self-reported ASD symptoms?

For the quantitative measures, Wilcoxon Signed Rank tests were used to assess potential changes in ASD knowledge, ASD symptoms, academic self-efficacy, and disability identity associated with participating in the program (see Table 3). Eta squared (η^2^) effect sizes were calculated. An increase in ASD knowledge (*Z* = −2.21, *p* = 0.03; η^2^ = 0.46) and a decrease in self-reported ASD symptoms was observed from pre- to post-test (*Z* = −2.14, *p* = 0.03, η^2^ = 0.49). *Post-hoc* analyses using nonparametric change score correlations revealed that change in ASD knowledge was not associated with change in ASD traits (*T* = 0.10, *p* = 0.71). No changes in disability identity or academic self-efficacy were observed (*p*s > 0.48).

**Table 3:** Wilcoxon signed rank tests of pre-post change in summer transition program 2.

**Measures**	**Pre-test (*N* = 10)**		**Post-test (*N* = 10)**		***Z, p***
	***M***	***SD***	***M***	***SD***	
ASD traits	67.30	28.45	62.40	24.80	*Z* = −2.14, *p* = 0.03
ASD knowledge	9.30	4.88	11.70	4.76	*Z* = −2.21, *p* = 0.03
Disability pride	11.10	4.28	11.80	3.82	*Z* = −0.71, *p* = 0.47
Feelings of exclusion	10.10	2.81	9.50	3.21	*Z* = −0.34, *p* = 0.73
Social model orientation	24.40	4.58	24.90	4.41	*Z* = −0.50, *p* = 0.62
Medical model orientation	20.10	3.78	19.80	4.10	*Z* = −0.33, *p* = 0.74
Self-efficacy	9.00	1.12	8.90	1.85	*Z* = −0.07, *p* = 0.94

*Significance values reflect findings from the Wilcoxon Signed Rank Test*.

The following trends were observed from pre- to post-testing on the qualitative measures. When asked to define self-advocacy at pre-test, eight out of nine students (one student was not asked this question) offered a definition, specifying speaking or standing up for yourself (*n* = 7), communicating effectively (*n* = 5), knowing your rights (*n* = 1), and doing things independently (*n* = 2). None of the students mentioned standing up for others. Students definitions of self-advocacy did not change substantively at post-test. The student who had said “*I don't know”* when asked what self-advocacy was at pre-test said “*I can't remember”* at post-test.

When asked at pre-test “In what ways has your disability made you a stronger person than you would have otherwise been?,” six out of nine students identified a strength. For example, it led to their interests (*n* = 2), e.g., “I think it's because of my fascination with numbers and also my fascination with philosophy and psychology…I like how the human brain works and also the human condition” and gave them a gift (*n* = 2), e.g., “Well it helped me to listen more and think more. It also gave me a deeper meaning to what it means to be social.” Students also noted that their disability afforded them an understanding of others (*n* = 2), made them unique (*n* = 1), and required them to work hard (*n* = 2). Three students were unable to identify strengths from their disability. At post-test, one student who had previously said “I don't know” when asked to identify a strength associated with his disability now said “It taught me what not to do.” A student who had said “I have no clue at the moment” at pre-test said “I can't answer that because it's personal” at post-test. The student who had said at pre-test “It did not make me a stronger person in any way” maintained at post-test “It made me a weaker person.” This student later indicated that members of his family often made fun of him for being autistic and that he does not identify as autistic (despite a PDD-NOS label on paperwork he provided when enrolling in the program).

When asked at pre-test “*What factors will you consider when deciding whether or not to disclose in college?”* only four students indicated evaluating circumstantial factors to decide whether or not to disclose e.g., “*I'd probably wait a long time unless they say they have the same thing.”* Post-test responses reflected a greater level of understanding of the complexities underlying disclosure, as eight out of nine students described evaluating circumstantial factors to decide whether or not to disclose their disability, e.g., trusting the person.

When asked at pre-test to role-play asking for accommodations if they needed them in college, almost all of the students indicated that they would ask a professor before or after class or during office hours (*n* = 8; two students did not wish to do a role-play). Only five students provided a *justification* for seeking accommodations, “*I have AS, and here are my papers and here are my accommodations…First off, may I record, do I have permission to record the slides.”* Two students requested non-disability related resources only, “*Excuse me. Can I ask you something? I really need your help. I really need your help on…my Physics. I don't really know all those, all those chemicals. Do you have any resource I can use?”* At post-test, seven students provided a justification for needed accommodations and no students requested non-disability related resources.

When participants were asked at post-test what they gained from the program, eight participants reported that they learned general skills (e.g., “*useful information that will help you in the future”*) and four participants said they learned college-specific skills (e.g., “*what to prepare before college starts”*). Four participants reported that they learned about self-advocacy. One of these participants noted, “*the whole experience of like, you know, there's more than one person who has like this disability…we all have a voice and we're all in this together.”* Another student noted, “*I learned a little bit about my disability and I'm still learning how to stand up for myself.”*

As part of the post-test interviews, both mentors and mentees reported on areas of the program they would change or improve upon. Several mentees (*n* = 3) and mentors (*n* = 2) cited scheduling or logistical concerns, including involving more students (*n* = 4) and making the program longer (*n* = 3). Several mentees (*n* = 2) and mentors (*n* = 2) provided suggestions for making the program more engaging. For example, one mentor suggested administering a pre-program poll to learn more about participants' hobbies and interests and subsequently tailoring program materials and activities to students' specific interests. One mentor and one mentee suggested that certain aspects of the program are repetitive (e.g., data collection methods), and that this should be addressed in future programs. Three mentees suggested additional topic areas and activities for the program. For example, one mentee suggested that the group take a field trip to an autism related organization. Another student suggested that instruction in daily living skills (e.g., how to live in a dorm) should be incorporated.

#### Insights from follow-up interviews

Four mentees agreed to participate in follow-up interviews a semester after participating in the program; three students met in-person and one student replied via email. When asked to reflect on what he had learned from other students in the program, a student who was still in high school said, “*I learned there a lot of people out there who have the same interests like me, who think like me, I think that is something I should keep in mind as I grow up.”* He described using self-advocacy skills he learned during the program to “*talk to my speech teacher…about maybe switching up a group I was in because the kid I was with; I was feeling very uncomfortable with this kid. So we changed groups and now I have this other kid and I'm really glad to go to this group with this kid.”* He said that he had been applying studying techniques from the program by “*taking uh more notes in school. But I do them in a very unique way. Sometimes I'll just put a little drawing that I'll immediately recognize as this means that or I'll phrase something a secret way so I really know what I'm thinking about… like hieroglyphics.”* When asked if it was helpful to have mentors on the spectrum, he said, “*I mean it's not that they didn't help. I feel that I got the same amount from those who were not on the spectrum.”*

The student who responded via email wrote highly succinct responses. She had told us during the program that she had been “forced” to enter the program by her parents and did not want to be there. Over time, she warmed to the program. She wrote that the most important thing she learned from other people in the program was “*I guess that college people aren't all super-scary, hard to approach people.”* When asked how she had used skills learned in the program, she wrote “*I have used self-advocacy with help from the Accessibility office in my school”* and described applying a social strategy we had emphasized of waiting for a break in a conversation to enter. She suggested that future programs focus more on managing stress.

The student who expressed a consistently negative viewpoint on his disability stated that the program had “*helped you know just explain college a lot…I guess I learned a lot about like autism.”* He described applying self-advocacy; “*I talked with the professor about stuff that I had. I just like talked with them you know and got help more.”* He reported particularly enjoying the games. When asked if it was helpful to have mentors on the spectrum, he said “*None of them said they were. I saw all of them. Which one….Oh yeah that one guy who liked Lincoln Park who was like 24. I feel like they all had Asperger's. Everybody basically.”*

Another student described a session he had particularly enjoyed when asked what he had shared with peers, “*I thought classroom etiquette was the most important so I shared my perspectives on classroom etiquette. College students should learn to act courteously toward others- like in the video- know how to leave conversations and how to stay*.” He described having used the social strategy of “*how to enter conversations and being able to work together*.” When asked if it was helpful to have mentors on the spectrum, he said, “*Yes. It was helpful because mentors are useful obviously. I would call them resources.…Mentors really help a lot. They contribute to discussion to sharing with other students and I thought that was very helpful*.” Although phrased differently, his response aligned with the other mentees' responses in reflecting an appreciation for mentors in general, rather than a specific enjoyment of autistic mentors. When asked what he had gained from the program, he said, “*I'm learning about classroom etiquette, self-advocacy and even getting better at transition. One level to the next level…If I had to rate the program I would give it high 90s. It might be my most favorite program.”*

#### What is the feasibility of a participatory approach to educational programming for autistic college students?

Findings from mentor and mentee semi-structured interviews suggest that the program was beneficial for both autistic and non-autistic mentors, particularly in regards to self-advocacy. One autistic mentor said, “*I always like meeting other autistic people, because before… early March I believe* (he was very recently diagnosed)*, I had never met an autistic person in my life. And what I thought it would be like wasn't exactly what it was. It was different. It was better.”* When asked what the most important thing he learned from students was, he said: “*I remember one student saying he…wanted to be cured. He wanted to be normal like everybody else, but I think by the time the program was over he realized that being normal is basically a figment of our imaginations. So I learned that a negative thinker can be turned into a realistic one.”*

The other autistic mentor (who had helped develop the curriculum) reported that the most important thing she had shared with mentees was her, “*sense of independence and like self-knowledge…because…the most important thing* [in college] *is that you learn about yourself.”* She said that the most important thing she learned from mentees was “*Becoming a self-advocate…I knew self-advocacy existed, within myself, but I think it wasn't until [mentorship program] first started that I started, that I started to put a definition on it.”* She said that her favorite aspect of the program was that “*I was like reminded of why I joined this program, why it's so important to me and why it's so important to other people.”*

A mentor without ASD said that she'd learned that “*even though we're different people, we still feel the same, in a sense…whether we have disorders or not*.” She reported that being a mentor “*helped me a lot, lowering my anxiety. And improved my social skills*.” Another mentor without ASD noted:

*I learned how important it is for self-acceptance and self-awareness from the students in the program. I knew that this population was subject to stigma, but I was unaware of the importance, and also the intricate methods, of disclosure… I loved learning from the students. It was amazing to watch them learn and grow at their own pace, and, as much as it was a program to help them learn about themselves, I was able to connect with them and learn things about myself along the way. They gave me a much bigger appreciation for people with disabilities, and confirmed my belief that their disability has the power to make them unique but powerful in their own right*.

When asked to describe characteristics that define them as a mentor, autistic and non-autistic mentors converged on the idea of empathy. Autistic mentors also emphasized honesty. For example, one autistic mentor stated, “*I don't put a mask on. I just say things how it is. Like I literally obsess with objectivity. So I'm always trying in some way, shape, or form, to picture myself in someone else's shoes. That's probably the best trait I would have for this.”*

Differences in mentorship approaches between autistic and non-autistic mentors were apparent in the way mentors approached mentees. Mentors without ASD emphasized using anecdotal or academic knowledge of ASD to tailor their mentorship approach to individual needs. For example, one mentor without ASD noted, “*I feel like the background info you give on autism is very helpful…I feel like a lot more people need to know about that.”* Another mentor without ASD said, *“By researching autism, and its effects, I was able to understand why some students were unresponsive, and therefore was able to adjust my methods as a mentor so I could reach them in a way that they felt more comfortable…I used a lot of online research and knowledge from my previous psychology courses concerning people with developmental disabilities to understand the students that I was working with.”*

In contrast, autistic mentors emphasized adapting to individual differences through observation. One autistic mentor stated, “*I started to figure out how each person was. Some were very open; some were very reserved… I mean it wasn't hard to figure out, but I kind of learned how to gauge myself differently with each person*.” He viewed his recent diagnosis as a “*bittersweet medium of empowerment.”* The other autistic mentor stated, “*I know that autism is a form of disability, but you know what, I think I'm gonna use the more neurodiversity version of it…Everybody is different, but I think you know the most that we have in common is skills of communication and thinking*.”

Both autistic and non-autistic mentors described analyzing their social interactions to adapt to mentees but the starting point and process of adaptation differed. When asked how she used her social skills as a mentor, an autistic mentor stated, “*I would like to call my social skills as a cue button, like you know when to speak and like knowing what to say and how to say it. What is my cue? When can I come in? When should I, you know, not butt in?”* In contrast, a mentor without ASD stated that, “*The only social skill that I struggled with during mentoring was connecting with those students that preferred to work alone or in a more private manner. Those students did not really like my overtly exaggerated social skills and would sometimes either not respond to me or shut down until I began focusing on them in a more laid back manner.”*

Although autism was a guiding metaphor for both autistic and non-autistic mentors, whether or not mentors had ASD does not seem to have been immediately relevant to *mentees*. This difference in salience may have arisen because the identity of mentors as autistic or not was not highlighted; mentors were *not* asked to disclose (and typically did so in non-obvious ways) because choosing when and how much to disclose is a central principle of self-advocacy.

## Discussion

The current study employed a multi-step participatory approach to the development, implementation, and evaluation of an initial feasibility assessment and pilot-test of a summer transition program to help incoming and current autistic college students develop self-determination skills and adapt to college. Consistent with a participatory framework, this process was iterative and collaborative, with autistic college students providing feedback throughout the process and helping to implement the program. Results from the current study are preliminary. Findings suggest potential benefits of participatory transition programming for fostering self-advocacy and social skills while leadership skills, in particular, require more time to develop than more basic aspects of self-advocacy.

### What skills do autistic college students need help developing?

Generally speaking, findings supported the importance of fostering self-advocacy skills among autistic college students, although students exhibited substantial variability in their self-advocacy skills at pre-test. Although the majority of students in the first transition program were not clear on the definition of self-advocacy, participants in the second program entered the program with greater knowledge about self-advocacy. The first program included only students who had been referred to the program by their academic advisors who were all planning to attend the college where it was hosted. In contrast, the second program included only students who were *not* planning to attend the college where it was hosted. It is conceivable that families that effectively seek out services that are not provided at the college their child is enrolled in are likely to have sought out prior services and supports that helped their child foster self-advocacy.

Although students in the second program were aware of what self-advocacy is and that they should approach a professor to ask for accommodations, they had not fully considered the details underlying this process. For example, several students did not provide a justification for needing accommodations and/or asked for non-disability related resources during pre-test role-plays. Furthermore, students were generally unfamiliar with the process of evaluating whether or not to disclose before participating in the program. Together, findings indicate a need for transition programing to help autistic students understand and practice self-advocacy.

### Was participation in the transition program associated with enhanced self-advocacy skills?

Prior to their participation in the second transition program, no students specifically mentioned an interest in learning self-advocacy-related skills. However, almost half of the participants reported that they gained self-advocacy skills after participation in the program, suggesting that incoming autistic college students may not be aware of the self-advocacy skills required for success in college. Participants demonstrated enhanced self-advocacy-related knowledge (i.e., knowledge about autism) and skills (i.e., ability to evaluate the context dependence of disclosure) following participation in the second summer transition program. However, findings suggest that students need more prolonged programming (such as the less intensive programming available through our mentorship program during the school year) to help them develop leadership skills, a critical component of self-advocacy. Indeed, it has taken a number of years for a critical mass of autistic students to become comfortable taking on leadership roles as mentors and researchers in our mentorship program. This current term six autistic students are mentors in our year-round mentorship program.

### Were reductions in autism symptoms reported following program participation?

Findings indicated a significant decrease in self-reported ASD traits from pre- to post-test, although given the short-term and self-report nature of the study, it is questionable as to whether decreases in autism severity reflect meaningful and sustainable change. However, similar, short-term improvements in communication skills have also been observed among youth in the general population who participated in a week-long summer camp in nature without access to technology (Uhls et al., 2014). In addition, a meta-analysis of social skills interventions for autistic adolescents revealed that they reported far greater improvements in *understanding of social situations* relative to *social behaviors* (Gates et al., 2017). Therefore, it is promising that students reported improvements in social behaviors in the current study. Future research should examine if self-reported improvements are corroborated by behavioral observations and persist over time. Given that autistic students with a broad range of cognitive skills are taking college classes (Zager and Alpern, 2010), future research should explore how variation in autistic traits and intelligence affects the experiences, perspectives and responsiveness to treatment of autistic college students.

### How feasible is a participatory approach to program development for autistic students?

Findings suggest that autistic and non-autistic mentors felt empowered by the opportunity to share their perspectives and knowledge with newer students and that becoming a mentor helped them develop leadership skills. We examined potential differences in mentorship approaches between autistic and non-autistic mentors to understand if there are unique advantages that either group may bring to mentorship. Several noteworthy patterns emerged from this line of inquiry. Although autistic and non-autistic mentors converged in describing empathy as a core skill they exhibited as a mentor, autistic mentors described an individualized approach to mentoring rooted in an understanding of the uniqueness of each autistic person coupled with a tendency to develop strategies for connecting with others in a precise manner. In contrast, mentors without ASD utilized academic knowledge about ASD coupled with a more intuitive approach to adapting their social interactions. This pattern was also reflected in the mentors' explanations of ASD, with autistic mentors emphasizing individual differences and mentors without ASD recounting the diagnostic criteria. These differences likely helped the program in effectively addressing the myriad unique needs of each participant by making different mentors skilled at demonstrating and explaining different skills.

### Limitations and future directions

While this study provides preliminary evidence in support of participatory transition programming for autistic students, there were limitations in the data collection and study design processes. With respect to data collection, the lack of verification of diagnosis using gold-standard diagnostic measures is a significant limitation of this study and of a number of prior studies with autistic college students (e.g., Schindler et al., 2015; Ames et al., 2016; Gillespie-Lynch et al., 2017a,b; Hillier et al., 2018). Similar to prior interventions for college students (Gelbar et al., 2014; Pugliese and White, 2014; Schindler et al., 2015), the number of participants was small, participants varied substantially in their educational level, and the sample lacked racial and ethnic diversity (primarily in STP1). In our efforts to deliver a program that met the needs and interests of autistic college students, we included both transitioning and current college students in this research. Indeed, other research studies with this population included both transitioning and current students in their programs (e.g., Schindler et al., 2015). However, it is conceivable that there are fundamental differences between these two groups that were not assessed through our research. Future research should investigate which supports may be most helpful for these distinct populations and assess any differences in the specific needs of these populations.

Also with respect to data collection, reliance on participants' self-reports limits the generalizability of findings of this research and the extant body of research conducted with college students (Pugliese and White, 2014; Schindler et al., 2015; Ames et al., 2016; Gillespie-Lynch et al., 2017a; Roberts and Birmingham, 2017; Hillier et al., 2018). When we first conceived of STP1, we attempted to collect behavioral data via videotaped role-play scenarios. However, a number of students' expressed heightened anxiety surrounding being video-taped. We resorted to audio-recording role plays, but students still declined to participate in them. In order to more effectively reduce anxiety surrounding behavioral observations in future program evaluations, participants could be provided with an outline of the activities they will participate in during assessments around a month before the program begins so they have sufficient time to become accustomed to the proposed assessments. Future research should include a range of outcome measures such as self-reports, parent reports, and behavioral assessments, as well as follow-up assessments in naturalistic settings to examine if improvements generalize across time and settings.

With respect to study design, this research did not utilize a control or comparison group, limiting interpretation and the generalizability of findings to other autistic college students. Indeed, this is a limitation in other programmatic research for this population (e.g., Pugliese and White, 2014; Schindler et al., 2015) and future research should seek to include a comparison group. An additional design limitation was a lack of independent evaluation of outcomes. Specifically, program staff were responsible for contributing to all aspects of the research, from administering the instructional modules to collecting qualitative and quantitative data. While our decision to maintain consistency in program staff potentially facilitated students' comfort in participating in the numerous surveys and questionnaires conducted as part of this research, this limitation reflects, in part, a global lack of available campus financial resources and personnel with an expertise in autism; this is a shortcoming that is often present in research with this population on college campuses (Barnhill, 2016). Indeed, much of the research on programming for autistic college students lacks an independent evaluator, often utilizing the mentor-mentee interactions as contexts for data collection (e.g., Ames et al., 2016; Roberts and Birmingham, 2017). Barnhill (2016) provides several recommendations for addressing the lack of sufficient resources for developing and evaluating programs for autistic college students, including offering graduate students the opportunity to receive internship credit and/or partnering with external community agencies that may undertake this work. Additional research utilizing independent evaluators is necessary to investigate these options.

Generally speaking, limitations in data collection and design apparent across studies evaluating supports for autistic college students likely reflect additional constraints associated with conducting interventions in the college environment, such as the need to adapt interventions to students' busy lives and the limited availability of a large quantity of autistic students at a given institution who are willing to take part in an intervention. Despite these limitations, foundational research such as the research described in the current report is recommended when initially developing and adapting programming for autistic individuals to ensure that it meets the needs of the people it is designed to serve (Taylor et al., 2012).

In order to build on the current research and address these limitations, we believe that multi-site collaborations are needed to more systematically evaluate interventions. It would be particularly helpful to systematically compare the benefits of transition programming that has been developed with and without an authentic participatory framework, to investigate potential differential benefits of individualized vs. group-based supports, and to compare benefits of programming delivered in high school to programming delivered just prior to beginning college. The mothers of the two high school students who participated in our second transition program reported that students on the spectrum need time to develop and practice skills before implementing them. Therefore, it is likely that a transition model with individualized and group supports delivered in high school, prior to college, and in college (akin to the model developed by White and colleagues that focuses on individualized supports in high school and college) is likely to be beneficial. Based on experience with our mentorship program wherein some autistic students prefer one-on-one mentoring, some prefer group mentoring and some prefer a combination of the two, a module based approach to transition programming wherein students have opportunities to select between different types of support is likely to have the most social validity. A modular approach with individualized supports is likely to be particularly beneficial for the types of autistic college students who tend to be the most challenging to support with limited resources, such as those with co-occurring mental health challenges. Future research should also expand on the strategies used to help autistic college students adapt to the social demands of college as social skills interventions, which we used as a model for the social interaction modules in our program, may inadvertently reduce social authenticity and increase stigma (Bottema-Beutel et al., 2017).

Although the research described in this report is closer to a truly participatory approach to developing supports for autistic college students than prior research, a truly participatory approach to programming would involve autistic college students in *all* aspects of the research process (see Nicolaidis et al., 2011 for an example of a truly participatory autism research design). Although autistic students played a leading role in adapting and implementing programming, they played a limited role in program evaluation. In our ongoing research, a team of autistic college students is developing research questions and will play a leading role in analyzing data from our mentorship program. However, it has taken years for a sufficient number of autistic college students to transition into leadership roles in the program, which has impeded our ability to conduct a truly participatory study. It is also likely that these students will require substantial levels of training and ongoing support to help them contribute substantively to program evaluation. As mentioned previously, two of the mentees in the first summer transition program are now mentors/researchers in our mentorship program. When asked to provide feedback on earlier drafts of this manuscript as part of their independent study research, they indicated that they approved of it. However, they did not provide detailed feedback despite conversations about the value of critiquing research in order to improve upon it. One autistic student wrote “*I think it was really good, it could be a little clearer.”* The other autistic student, who is an honors student, wrote that it “*looked pretty legit.”*

Taken together, although the current research represents an important step for the field, the findings are preliminary and should be considered as such. Future research with autistic college students should build on and advance the current research by addressing the gaps and recommendations for rigorous participatory research outlined above.

## Conclusions and reflections

This study addresses an important gap in the literature regarding transition programing for autistic college students by developing a process through which more experienced autistic college students can play a leadership role in fostering self-advocacy skills among autistic students who are transitioning into or struggling in college. This research suggests that participation in a brief but intensive summer transition program may help prepare autistic college students to self-advocate and engage with diverse peers in college contexts. By engaging current autistic college students in the process of developing, implementing and evaluating supports for newer autistic students, educators can empower autistic students to take on leadership roles while developing programming that is rooted in the lived experience of being an autistic college student. This type of approach is likely to enhance the social validity of programming by drawing upon strengths associated with autism that are likely to be helpful when evaluating programming, such as heightened attention to detail and honesty.

By using a participatory approach to developing transition supports for autistic students, we can provide opportunities for current autistic college students to develop skills and experiences that will be useful when seeking employment while demonstrating to newer autistic students that they can also become leaders in the future. Students made several suggestions for enhancing transition programming further, including providing a longer program with opportunities for field trips and continuing to develop programming that aligns with students' interests and needs by incorporating more needs assessments and hands-on interactive activities. Future programming should expand on the findings from the current study by incorporating these and other programming recommendations from autistic college students.

## Ethics statement

This study was carried out in accordance with the recommendations of the Human Research Protections Program at the College of Staten Island and Hunter College with written informed consent from all subjects. All subjects gave written informed consent in accordance with the Declaration of Helsinki. The protocol was approved by the Human Research Protections Programs at the College of Staten Island and Hunter College.

## Author contributions

All authors made substantial contributions to: (1) the conception or design of the work or the acquisition, analysis, or interpretation of data for the work; (2) drafting the work or revising it critically for important intellectual content; and (3) final approval of the version to be published. All authors agree to be accountable for all aspects of the work in ensuring that questions related to the accuracy or integrity of any part of the work are appropriately investigated and resolved. Specific contributions: EH and CS-S collaborated on the initial development of the summer transition program, program implementation, data collection, and data coding and analysis. EH also led the drafting of the current manuscript. RO, DD, MS, CC, JP, AM, MG, and JD contributed to program development and implementation. This research was guided by KG-L, who developed the original idea for this study, played a guiding role in program and assessment design, and contributed very substantially to the final manuscript.

### Conflict of interest statement

The authors declare that the research was conducted in the absence of any commercial or financial relationships that could be construed as a potential conflict of interest.

## Footnotes

^1^The term “autistic person” is preferred throughout this report rather than “person with autism” in order to respect the preferences of autistic people who often prefer “identity first” terms over “person first” terms (e.g., Kapp et al., 2013).

^2^Individuals with Disabilities Education Act, 20 U.S.C. § 1400 (2004).

^3^The ADA Amendments Act of 2008. PUBLIC LAW, 110, 325.
