# Supplementary material for: Designing a Summer Transition Program for Incoming and Current College Students on the Autism Spectrum: A Participatory Approach
Source: Front Psychol. 2018 Feb 13;9:46. doi: 10.3389/fpsyg.2018.00046 (PMC5816926; doi:10.3389/fpsyg.2018.00046)
Supplement: Supplementary file 1 [file Table1.pdf]

Appendix A: Syllabi from Summer Transition Programs 1 and 2

Summer Transition Program 1
[COLLEGE]
Department of Psychology and Center for Student Accessibility

Instructor One: [NAME]
Email: [E-MAIL ADDRESS]

Instructor Two: NAME]
Email: [E-MAIL ADDRESS]

Class Location: [CLASS LOCATION]
Meeting Time: August 18th-21st from 12pm-5pm each day; August 22nd from 12pm-3pm

Course Objectives: There are four main areas of focus for this program that include developing social skills, self-advocacy skills, classroom readiness skills, and technology skills. All of these have been identified as important skills to develop for the first year of college.

Readings: Hand-outs and articles will be assigned throughout the week. All additional readings and assignments will be uploaded to Blackboard.

Student Privacy Statement:
At times, students may disclose personal information through workshop discussions. It is expected that the class will respect the privacy of their classmates. The information disclosed in this class should not be repeated or discussed with other students outside of the course.

How to do well in this program:
- ✓ COMPLETE any mini-homework assignments before each class.
- ✓ OUTLINE or take notes on the lectures and readings.
- ✓ PARTICIPATE in class discussions, activities, and engage in critical thinking.
- ✓ CHECK Blackboard regularly for announcements, assignments, discussions and more.

Participation & Attendance: Participation involves listening to peers', contributing to the discussions online and in-person, and asking questions during the workshops. All phones should be turned off and put away, and absolutely NO TEXTING is permitted during the program.

All Students are expected to attend all workshop dates. If you miss a workshop day, you should find out what you missed from another student in the workshop.

Constructive Feedback: There will be many opportunities for you to receive feedback from the instructors, program volunteers, and your peers. In addition, you'll be expected to provide constructive feedback to your peers. You should be giving feedback to others that can help build them up in a way that lets them know you're on their side. Your feedback should not put people down, but should instead help people to improve their skills.

WORKSHOP SCHEDULE

*Please note that this is a tentative schedule that it is subject to change.

Date	Topics	Assignment Due
Mon.<br>8/18	Introduction Activity & Group Promises	
	Syllabus Overview	
	Classroom Readiness: Learning Styles & Study Skills	
	Leadership Game	
	Social: Reading Body Language	
	Social Skills: Friendships	
	Campus Resources	
	Advocacy Skills: Sensory Preferences	
Tues.<br>8/19	Classroom Skills: Blackboard	Email a Professor Assignment
	Advocacy: Intro.	
	Classroom Skills:<br>Leadership & Public Speaking	
	Advocacy Skills:<br>ASD	
	Advocacy Skills:<br>Accommodations	
	Social: Self-Regulation	
Wed.<br>8/20	Social Skills: Facebook and Email	
	Presentation Work	
	Social Skills: Conversations	
	Social Skills:	

	Resolving Disagreements	
	Advocacy Skills:<br>Identifying Laws	
	Campus Resources:<br>Scavenger Hunt	
Thurs.<br>8/21	Social: Small Talk	Email Your Professor<br>Assignment (part II)
	Social Skills:<br>Social Networking Sites	
	Classroom Skills:<br>Classroom Etiquette	
	Classroom Skills:<br>Note-Taking & Planning	
	Presentations and Party	

Use of Blackboard: Students in this training are required to use Blackboard. It is your responsibility to check Blackboard regularly (at least once a day) for announcements and assignments. It is essential that you are able to receive emails through blackboard as that will be the primary means for out-of-class communication.

Blackboard and Email
For many assignments and communications Blackboard will be the medium of communication. Please make sure you can log into this course on Blackboard.

Please understand I receive many emails each day. To ensure your email is seen, read, and receives a timely response use the following format for all email communications with me:

> To: [E-MAIL ADDRESS]
> Subject: SUMMER TRANSITION YOUR LAST NAME (e.g. Smith) HOMEWORK ASSIGNMENT (OR any brief relevant words about the reason for your email)
> *Sign your FULL NAME to the email

Summer Transition Program 2
[NAME] College
Department of Psychology

Instructors: [NAMES]
Emails: [E-MAIL ADDRESSES]
Class Dates: August 3rd – August 7th
Class Locations: [CLASS LOCATION]

Course Description: This summer transition program will cover multiple skill sets that are believed to be useful in the college environment. Each day will include a variety of sessions that tackle different topics (e.g., self-advocacy, maintaining friendships, effective habits for engaging in small talk, etc.). These topics will be studied through Powerpoint Presentations, in-class activities such as scripted role-play's or small group tasks, games and interactive opportunities, and media clips. Throughout the week, instructors will provide students with opportunities to learn more about each topic, practice skills, and receive feedback on their application of these skills.

Course Learning Objectives: Students will learn how to define, evaluate and develop their skills in four broad domains:

Academic readiness skills: Students will practice identifying their learning styles, taking notes, and engaging productively in class activities

Self-advocacy skills: Students will practice defining, engaging in and evaluating self-advocacy

Self-regulation skills: Students will practice identifying and evaluating self-regulation strategies

Social skills: Students will practice engaging effectively with peers and instructors in the college environment

Program Requirements:
- Writing utensil and paper
- Binder for program materials
- Navigating College Handbook: This will be provided to each student on the first day.

Student Privacy Statement:
At times, students may disclose personal information through workshop discussions. *Students should never feel obliged to disclose anything they do not feel comfortable sharing.* Although all participants are expected to respect the privacy of their peers by not disclosing confidential information to people outside of this program, disclosure is always associated with the possibility of a breach of confidentiality. Therefore, each participant should only disclose as much personal information as he or she feels comfortable disclosing.

How to do well in this program:
- ✓ COMPLETE all assignments each day.
- ✓ OUTLINE or take notes on all lectures and readings.
- ✓ PARTICIPATE in class discussions, activities, and engage in critical thinking.

Participation & Attendance: Participation involves listening to peers', contributing to discussions online and in-person, and asking questions. All phones should be turned off and put away, and absolutely NO TEXTING is permitted in the classroom. If you have an important communication to attend to, please excuse yourself from the classroom and attend to the communication in the hall. Leaving the classroom frequently is disruptive for other students so be sure to limit the amount of time you spend outside of the classroom during class activities. Students are expected to attend all programming dates. If you miss activities, you should find out what you missed from other students in the workshop.

Constructive Feedback: There will be many opportunities for you to receive feedback from the instructors, program volunteers, and your peers. In addition, you'll be expected to provide constructive feedback to your peers. You should be giving feedback to others that can help build them up in a way that lets them know you're on their side. Your feedback should not put people down, but should instead help people to improve their skills.

Evaluation: You will not be graded for your participation in this program as we would like for you to demonstrate intrinsic motivation.
However, you will receive written feedback on at least one key activity each day including:
a. Your notes from two class activities *On Tuesday and Thursday
b. In-class writing/computer-based assignments * One each day
c. A presentation you create and share *On the last day of the program

SCHEDULE
*Please note that this is a tentative schedule that is subject to change.

Date	Topics	Assignment Due
Mon. 8/3/15	Pre-Assessments	
	Game & Introductions	
	Reading a Syllabus & Semester Planning	
	Introduction to Self-Advocacy & Daily Jeopardy Review	Self-Check Quiz
Tues. 8/4/15	Game & Classroom Etiquette	Writing Assignment
	Self-Regulation	Writing Assignment
	Lunch & Game	
	Embracing Social Opportunities in College	Writing Assignment
	Leadership & Public Speaking	

	Campus Scavenger Hunt, Review Self-Regulation, & Daily Jeopardy Review	Self-Check Quiz
Wed. 8/5/15	Game & Engaging in Small Talk	
	Entering & Exiting Conversations	
	Autism Knowledge & Note-Taking	Writing Assignment
	Lunch & Game	
	Learning Styles & Study Skills	
	Disclosure & Speech Practice Time	
	Asking for Accommodations & Daily Jeopardy Review	Email Writing Assignment; Self-Check Quiz
Thurs. 8/6/15	Game & Reading Body Language	
	Sensory Preferences	
	Lunch & Game	
	Maintaining Friendships	
	Writing Skills	
	Emailing Your Professors & Daily Jeopardy Review	Email Writing Assignment; Self-Check Quiz
Fri. 8/7/15	Final Jeopardy Review	
	Public Speaking Activity	
	Pizza Party & Post-Assessments	

Email Communication

Please understand that your instructors receive many emails each day. To ensure that your email is seen, read, and receives a timely response use the following format for all email communications with the instructors:

To: [EMAIL ADDRESS]
Subject: SUMMER TRANSITION YOUR LAST NAME (e.g. Smith) HOMEWORK ASSIGNMENT (OR any brief relevant words about the reason for your email)
*Sign your FULL NAME to the email
